# New Gel-Like Polymers as Selective Weak-Base Anion Exchangers

**DOI:** 10.1371/journal.pone.0122891

**Published:** 2015-05-06

**Authors:** Błażej Gierczyk, Michał Cegłowski, Maciej Zalas

**Affiliations:** Faculty of Chemistry, Adam Mickiewicz University in Poznań, Poznań, Poland; Reader in Inorganic Chemistry, UNITED KINGDOM

## Abstract

A group of new anion exchangers, based on polyamine podands and of excellent ion-binding capacity, were synthesized. The materials were obtained in reactions between various poly(ethyleneamines) with glycidyl derivatives of cyclotetrasiloxane. The final polymeric, strongly cross-linked materials form gel-like solids. Their structures and interactions with anions adsorbed were studied by spectroscopic methods (CP-MAS NMR, FR-IR, UV-Vis). The sorption isotherms and kinetic parameters were determined for 29 anions. Materials studied show high ion capacity and selectivity towards some important anions, e.g., selenate(VI) or perrhenate.

## Introduction

The search for selective polymers or hybrid exchangers or sorbents of anionic species is a fast-developing aspect of material chemistry [1,2,3,4], however the number of reports on this topic is much lower than those on the sorption of cations by polymer materials. The anion-binding and anion-exchanging polymers and composites are of interest as materials for harmful anion removal from drinking water [5,6,7], waste-water management [8,9,10], analyte preconcentration prior to anionic-species analysis [11,12], as carriers of anionic pharmaceuticals [13,14,15] or for catalysts recovery [16]. A variety of materials have been tested for anion sorption, including the inorganic sorbents (such as alumina and other metal oxides) [6,17,18], synthetic resins [19,20], chitosan [5,7,21,22,23] and a wide range of inorganic and organic supports grafted with anion binding side-chains [11,12,24,25]. In most cases, the active anion-binding sites are the quaternized amino groups (both exo- and endocyclic), which interact with anions through electrostatic forces. For some oxoanions, more specific systems have been designed, e.g. 1,2-diaminoaryl moieties for Se(IV) separation [12] or organophosphonate-Zr(IV) adducts for binding of tungstate(VI) and molybdate(VI) [26].

In our previous paper we have shown the preparation and application of novel gel-like polyamine resins for removal of heavy metal cations [27]. The synthesis of the polymer involves a new strategy, based on simultaneous polymerization of polysiloxane precursor and its modification by chelating units. We decided to use the same material for preparation of anion-exchange polymers, containing ionized amino groups, i.e. weak-base ion exchangers. This class of ion-exchange materials has some important advantages over strong-base ones (materials containing quaternized ammonium and phosphonium centres). The most important one is the increased selectivity towards some oxyanions. Strong-base exchangers are hydrophobic, therefore the major parameter determining their selectivity towards various anions is the degree of the hydration of separated ions, as long as their charge remains the same. The weak-base exchangers interacts with anions adsorbed not only via the electrostatic forces, but by formation of hydrogen and coordination bonds. This change their selectivity, e.g. increases the affinity to multivalent anions or oxoanions containing larger number of oxygen atoms [28–31].

## Experimental

All reagents used are commercial products. The salts used for anion binding studies had the purity grade p.a. or higher and were purchased from Sigma-Aldrich. They were used without purification. The starting polymers (**N2**-**N6**) were obtained according to procedures described earlier [27]. All other compounds were purchased from Sigma-Aldrich and Merck and used as received. The solvents used for measurements were SPECTRANAL grade.

### Synthesis of protonated resins

One g of the starting polymer (**N2**-**N6**) was ground in a mortar and suspended in acetonitrile (50 ml). To the obtained mixture, fivefold molar excess (calculated on the basis of the nitrogen amount in the starting material) of perchloric or hexafluorophosphoric(V) acid in acetonitrile (5% by weight) was added. The suspensions obtained were mixed overnight, then the solid resins were filtered off, washed three times with acetonitrile and dried in vacuum at room temperature.

#### CAUTION

Solutions of perchloric acid in organic solvents may be explosive. Amine perchlorates could detonate upon heating or hitting.

### Spectroscopy

Nuclear magnetic resonance spectra of the solids (^13^C, ^15^N, ^29^Si) were obtained at 298 K, on a Varian VNMR-S 400 MHz spectrometer, operating at frequencies 101.25, 40.84 and 80.00 MHz, respectively. The standard CP-MAS technique was used (*tancpx* sequence with ^1^H decoupling). The ^13^C spectra were referred to the methylene signal of external glycine (43.30 ppm), acquisition time—40 ms, cross-polarization contact time—2 ms, sample rotation—5 kHz. The ^15^N spectra were referred to the signal of external glycine (-347.60 ppm), acquisition time—35 ms, cross-polarization contact time—2 ms, sample rotation—6 kHz. The ^29^Si spectra were referred to external neat TMS (0.00 ppm), the acquisition parameters were the same as for ^15^N NMR spectra.

Infrared spectra were recorded on a Bruker IFS 66s spectrometer, using KBr pellets (2 mg of sample in 200 mg of KBr). The complexes for these measurements were prepared by immersion of a polymer sample in 1·10^–2^ M solution of the corresponding sodium salt in water for 10 h, followed by decantation, washing of a solid material with water and methanol and drying in vacuum at room temperature.

### Elemental analysis, chemical analysis

The contents of C, H and N were determined using a Vario EL III elemental analyser. The content of silicon in the polymers was determined on an EDXRF spectrometer MiniPal 2 (PANalytical) after sample solubilisation with hydrofluoric acid. Iodine and phosphorus content in final polymers, after its solubilisation in 0.5 M NaOH, were determined by the ICP-OES method on a Varian Vista-MPX spectrometer with argon plasma ionization. The perchlorates were determined in the same solution spectrophotometrically, as an ion-pair with Brilliant Green according to the procedure described by Fogg et al [32]. Ion-chromatographic analyses were carried out on Methom Vario system.

### Isotherms of adsorption, adsorption kinetics, sorption-regeneration experiments

The adsorption measurements were performed for water solutions of sodium salts (except tellurate, which was used as a potassium salt) of studied ions (F^-^, Cl^-^, Br^-^, I^-^, NO_3_^-^, NO_2_^-^, ClO_3_^-^, ClO_4_^-^, BrO_3_^-^, SO_3_^2-^, SO_4_^2-^, SeO_3_^2-^, SeO_4_^2-^, TeO_3_^2-^, TeO_4_^2-^, PO_4_^3-^, HPO_3_^2-^, H_2_PO_2_^-^, P_2_O_7_^4-^, P_3_O_9_^3-^, AsO_2_^-^, HAsO_4_^2-^, MnO_4_^-^, ReO_4_^-^, CrO_4_^2-^, WO_4_^2-^, MoO_4_^2-^, VO_3_^-^ and VO_4_^3-^). To make each isotherm, a series of samples containing 0.01 g of polymer and 5 ml of salt solution were used. The adsorption properties at eight concentrations were measured for each system (0.1, 0.5, 1, 2, 3, 4, 5 and 10 mM). The pH of the solutions was adjusted with NaOH and HClO_4_ or HPF_6_ (depending on the polymer counter ion) to 5.0. The mixtures were equilibrated for 24 h at 298 ± 1 K. The starting (*C*_*0*_) and final, equilibrium (*C*_*A*_) concentrations of anions were determined with the method dependent on the character of the counter ion in the polymer (Table 1). The amount of the anion adsorbed (*q*_*A*_; mmol/g) in equilibrium was calculated from the difference between these concentrations (Eq 1):

$$q_{A}=\frac{\left(C_{0}-C_{A}\right)V}{m}$$ 1

where *m* is the mass of the adsorbing polymer (g) and *V* is the volume of salt solution (dm^3^).

**Table 1:** Methods used for determination of the anion concentration dependent on the counter-ion in polymer.

Anion adsorbed	Analytical method	
	ClO_4_-containing resin	PF_6_-containing resin
F^-^	potentiometry	potentiometry
Cl^-^	argentometry	XRF
Br^-^, BrO_3_^-^	XRF	XRF
NO_3_^-^, NO_2_^-^	ion chromatography	ion chromatography
ClO_3_^-^	ion chromatography	XRF
ClO_4_^-^	-	XRF
I^-^, SO_3_^2-^, SO_4_^2-^, SeO_3_^2-^, SeO_4_^2-^, TeO_3_^2-^, TeO_4_^2-^, AsO_2_^-^, HAsO_4_^2-^, MnO_4_^-^, ReO_4_^-^, CrO_4_^2-^, WO_4_^2-^, MoO_4_^2-^, VO_3_^-^, VO_4_^3-^	ICP-OES	ICP-OES
PO_4_^3-^, HPO_3_^2-^, H_2_PO_2_^-^, P_2_O_7_^4-^, P_3_O_9_^3-^	ICP-OES	ion chromatography

The relations between the sorption properties of the polymers studied and the pH of the solution were studied in various pH ranges, taking into regard the complex pH-dependent equilibriums known for some anions studied (e.g. MoO_4_^2-^ or WO_4_^2-^) or anion instability (e.g. SO_3_^2-^). The pH of the solutions was adjusted with NaOH and HClO_4_ (or HPF_6_). To calculate the maximal amount of the metal ions adsorbed by the polymer, the measurements were made for five salt concentrations. Other experimental details were as for the isotherms determination.

The adsorption kinetics was studied for 1 × 10^–2^ M salt concentration, using 20 mg of finely ground adsorbent (150–200 mesh) and 20 ml of adsorbate solution at 298 ± 1 K; pH was adjusted at 5.0. This stoichiometry guarantees large excess of the ions over the binding sites, therefore the pseudo second-order kinetic model could be used. The reaction mixtures were magnetically stirred vigorously at a stirring speed of 800 rpm. The same volume aliquots (0.1 ml) were withdrawn periodically and the metal concentration was determined by the methods mentioned above. The first-order rate constants were calculated from the equation (Eq 2):

$$\text{ln}\left(q_{A}-q_{t}\right)=\text{ln}\;q_{A}-k_{1}t$$ 2

while the second-order rate constants were calculated from the equation (Eq 3):

$$q_{t}^{-1}={\left(k_{2}q_{A}\right)}^{-1}t^{-1}+q_{A}^{-1}$$ 3

where *q*_*t*_ and *q*_*A*_ are the amounts of anion adsorbed after time *t* and at equilibrium, respectively, (in mmol of adsorbate per 1 g of sorbent); *k*_*1*_, *k*_*2*_ are the rate constants.

Sorption-regeneration experiments were made for 0.05 g samples of resins. The sample studied was suspended in 5 ml of the appropriate adsorbate solution (1 × 10^–2^ M), pH was adjusted to 5.0 and the suspension was stirred vigorously for over 5 h. Then the solid material was filtered off. The amount of exchanged ions was determined in the filtrate by the method indicated in Table 1. The resins were regenerated in two ways. The first method was a two-step process. The solid material was suspended in 10 ml of NaOH solution (1 × 10^–2^ M), mixed over 5 h, separated by centrifugation, washed with water and protonated with HClO_4_ (or HPF_6_) as described in section 2.1. The amount of desorbed ions was determined in alkaline supernatant. The second method of resins regeneration consisted of mixing it with 10 ml of 10% solution of NaClO_4_ (or NaPF_6_) and separation of the solid material by centrifugation. The resins were re-used after washing with water. The concentration of desorbed ions was determined in brine.

Ten cycles of ions binding/sorbent regeneration were performed. The efficiency of the anion retention in the *n*-th cycle was expressed as the retention factor *R*_*n*_ (Eq 4):

$$R_{n}=\frac{n_{n}^{A}}{n_{1}^{A}}$$ 4

while the ability to ions release in alkaline/brine solution in the *n*-th cycle was described by the release factor (Eq 5):

$$D_{n}=\frac{n_{n}^{R}}{n_{1}^{A}}$$ 5

where *n*_*1*_^*A*^, *n*_*n*_^*A*^ are the amounts of adsorbed ions in the first and *n*-th adsorption cycle (in mmol), *n*_*n*_^*R*^—the amount of anions released during the *n*-th regeneration.

### Water uptake

The swelling of the resins was studied in water. The polymers used for this study were vacuum dried at 373 K and the particles of 20–50 mesh were used. The dry, weighted material (about 250 mg) was allowed to swell in water for 24 h at 298 K, filtered off, the excess of water was removed by blotting and the sample was weighted again. The water uptake was calculated from equation (Eq 6):

$$W=\frac{m_{s}-m_{0}}{m_{0}}$$ 6

where *m*_*0*_ and *m*_*s*_ are the masses of dry and swelled samples respectively.

### Thermal analysis

Derivative scanning calorimetry and thermogravimetric measurements were performed on a Setoram TGA thermoanalyser in nitrogen atmosphere at the constant heating rate of 10 K min^-1^. The samples were dried in vacuum (1 mmHg) at 353 K for 5 h before analysis. Due to violent decomposition of perchlorate-containing resins, only hexafluorophosphate(V) derivatives were studied.

## Results and Discussion

### Polymer structure

**Fig 1:**
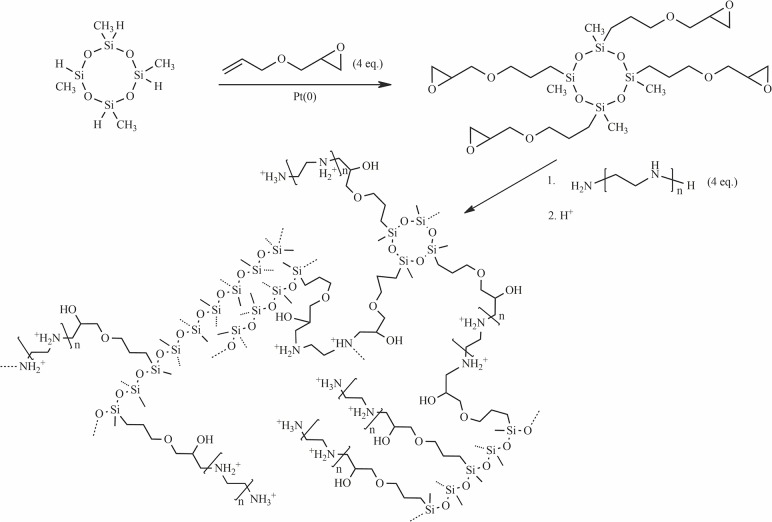
Synthetic route to studied ion-exchange resins.

The new polymeric ion adsorbents with polyamine podand arms were synthesized according to the synthetic route presented in Fig 1. After formation of cross-linked polyamine/polysiloxane resin, the nitrogen atoms were quaternalized by protonation with perchloric or hexafluorophosphoric acid. These acids were chosen due to their chemical properties. Both are strong, monoprotonic acids (pK_a_ < -5), therefore they do not undergo anionic hydrolysis, which may influence the sorption properties in more acidic solution. Perchlorate anion forms strong hydrogen bonds with polyamine net of the studied materials and is effectively bonded by them. Contrary, PF_6_^-^ anion is weakly adsorbed and easily exchanged. The use of these anions as counter-ions in the resins studied permits the studies and comparison of the sorption of both weakly adsorbed anions (e.g. halides) as well as strongly bonded ones (e.g. perrhenate, selenate(VI)). Moreover, they are stable in oxidizing and reducing conditions, therefore they do not react with anions used for the sorption studies (e.g. permanganates or hypophosphites). Finally, both show strong bands in IR spectra, which allows monitoring of their exchange by this technique. The elemental compositions of the polymers and their structural features are presented in Tables 2 & 3. The amount of protonated nitrogen atoms was calculated from elemental analysis and N^1^:N^2^:N^3^ ratio in parent polymers. As shown for all studied systems, the cationized nitrogen atoms constitute over 90% of the total number of nitrogen atoms.

**Table 2:** Elemental analysis data and yields of the resins studied and the parent polymers.

		Elemental composition [%]					
Symbol^a^	Amine	H	C	N	Si	Cl	P
**N2**	H_2_NCH_2_CH_2_NH_2_	9.22	46.43	10.14	12.59	-	-
**N2HCl**	H_2_NCH_2_CH_2_NH_2_	5.77	26.93	5.92	7.30	14.50	-
**N2HP**	H_2_NCH_2_CH_2_NH_2_	4.88	22.64	4.99	6.15	-	10.85
**N3**	H_2_N(CH_2_CH_2_NH)_2_H	9.68	47.76	13.43	10.75	-	-
**N3HCl**	H_2_N(CH_2_CH_2_NH)_2_H	5.50	24.61	7.00	5.69	17.20	-
**N3HP**	H_2_N(CH_2_CH_2_NH)_2_H	5.24	19.99	5.63	4.60	-	12.19
**N4**	H_2_N(CH_2_CH_2_NH)_3_H	9.94	48.62	15.68	9.47	-	-
**N4HCl**	H_2_N(CH_2_CH_2_NH)_3_H	5.29	23.01	7.47	4.73	18.50	-
**N4HP**	H_2_N(CH_2_CH_2_NH)_3_H	5.44	18.49	6.01	3.67	-	13.06
**N5**	H_2_N(CH_2_CH_2_NH)_4_H	10.08	49.40	17.70	8.34	-	-
**N5HCl**	H_2_N(CH_2_CH_2_NH)_4_H	5.05	21.99	7.82	3.76	19.63	-
**N5HP**	H_2_N(CH_2_CH_2_NH)_4_H	5.65	17.42	6.30	3.03	-	13.64
**N6**	H_2_N(CH_2_CH_2_NH)_5_H	10.35	50.11	19.22	7.57	-	-
**N6HCl**	H_2_N(CH_2_CH_2_NH)_5_H	5.00	21.16	8.14	3.22	20.20	-
**N6HP**	H_2_N(CH_2_CH_2_NH)_5_H	5.77	16.77	6.46	2.50	-	14.08

^a^HCl—perchlorate, HP—hexafluorophosphate(V)

**Table 3:** Results of the chemical analysis of the polymers studied.

Polymer	Parameter			
	N^1^:N^2^:N^3^	N:Cl or N:P	N^+^	WA [%]
**N2**	0.373:0.621:0.006	-	-	0.30
**N2HCl**	-	1.035	0.966	0.61
**N2HP**	-	1.018	0.982	0.55
**N3**	0.261:0.731:0.008	-	-	0.48
**N3HCl**	-	1.032	0.969	0.76
**N3HP**	-	1.023	0.977	0.70
**N4**	0.193:0.798:0.009	-	-	0.65
**N4HCl**	-	1.024	0.977	1.03
**N4HP**	-	1.019	0.981	0.96
**N5**	0.163:0.830:0.007	-	-	0.77
**N5HCl**	-	1.010	0.990	1.25
**N5HP**	-	1.023	0.978	1.20
**N6**	0.136:0.854:0.010	-	-	0.91
**N6HCl**	-	1.022	0.979	1.40
**N6HP**	-	1.016	0.984	1.30

N^1^:N^2^:N^3^—ratio of primary:secondary:tertiary nitrogen atoms in polymer; N:Cl or N:P—moles of counter ion (ClO_4_^-^ or PF_6_^-^) per 1 mol of nitrogen atoms; N^+^—molar ratio of protonated nitrogen atoms to total nitrogen; WA—water uptake

Water uptake by the resins studied was high. The values of this parameter were distinctly higher than that for the parent, non-ionic polymers [27]. This is a result of higher affinity of water to cationic N-centres than to amino groups as well as high hydration rates of perchlorate and hexafluorophosphate(V) ions. For the resins studied, the water uptake increases with increasing polyamine chain length, which is a result of an increasing number of water binding groups (N atoms and counter ions) as well as loosening of the polymer structure due to elongation of the cross-linking chains.

The materials obtained are stable in acidic conditions—i.e. the polymer matrix does not show any signs of decomposition after stirring for 10 days in 1 M HCl or H_2_SO_4_. The IR and NMR spectra of so treated resins are identical as those of the parent ones (except changes resulting from ion-exchange). The treatment of the exchangers with strong, concentrated base solutions (e.g. 1 M NaOH or KOH) leads to fragmentation of poly(siloxane) chains and decomposition of the polymers studied. They are however stable in less concentrated hydroxide solutions (< 0.05 M).

### CP-MAS spectra of polymers and their complexes

The structures of the polymers obtained are fully confirmed by NMR measurements. The ^13^C spectra are very similar to those of parent resins. The signals of non-polyamine part of the molecules were observed at the same chemical shifts in both protonated and neutral polymers [27]. The siloxane parts of the molecules gave the characteristic signals at -1.7 ppm (Si***C***H_3_, cyclic siloxane), -0.1 ppm (Si***C***H_3_, linear siloxane), 13.6 ppm (Si***C***H_2_), 23.5 ppm (C***C***H_2_C), 68.8 ppm (***C***HOH) and 74.0 ppm (***C***H_2_O). The only difference was the greater line widths observed for the cationic forms. The ionization affects strongly the signals of the carbon atoms bonded to nitrogen atoms. Protonation resulted in the 3–7 ppm upfield shift of the signals assigned to the nitrogen-bonded C atoms. The ^29^Si NMR spectra of the polymers studied were not affected by protonation of polyamine part of the molecule, i.e. the signals appeared at -21.8 (linear polysiloxane) and -16.5 ppm (cyclotetrasiloxane). The ^15^N NMR spectra of protonated polymers show the signals at ca. -336, -338 and -325 ppm (traces), corresponding to R_2_NH_2_^+^, RNH_3_^+^ and R_3_NH^+^ centres. Anion exchange does not affect the ^13^C, ^15^N and ^29^Si NMR spectra, i.e. the observed shifts are smaller than the measurements accuracy. As the signals of the studied resins are rather broad, shifts smaller than 2 ppm are not detectable. For the complexes with CrO_4_^2-^, a large increase in the NMR line widths was detected, caused by paramagnetic relaxation induced by Cr^3+^ ion, formed by reduction of chromate(VI) anions. The NMR data are collected in Table 4.

**Table 4:** NMR data for the polymers studied.

Sample	Chemical shift [ppm]								
	^13^C NMR							^29^Si NMR	^15^N NMR
	*C*H_3_	*C*H_2_Si	*C*H_2_ CH_2_Si	O*C*H_2_	*C*HOH	CH*C*H_2_N	CH_2_N		
**N2Cl**	-1.5; -0.2	13.4	22.1	74.2	68.9	56.7	37.5–53.0	-22.0	-336.2; -337.7
**N3Cl**	-1.9; -0.1	13.7	23.6	74.3	68.9	54.2	37.1–52.0	-16.5; -22.1	-336.1; -337.5
**N4Cl**	-2.0; -0.1	13.5	24.1	74.2	69.0	56.0	36.5–52.5	-16.9; -22.0	-325.5; -336.1; -337.9
**N5Cl**	-1.6; 0.0	13.8	24.0	74.3	69.1	56.6	37.0–52.5	-16.9; -22.1	-336.2; -337.4
**N6Cl**	-1.5; -0.1	13.8	24.1	74.4	68.7	56.1	37.6–53.0	-16.8; -22.2	-325.7; -336.0; -337.7
**N2P**	-1.4; -0.1	13.0	22.6	74.6	69.4	56.4	38.0–53.0	-21.9	-336.0; -337.7
**N3P**	-1.5; -0.1	13.6	23.3	74.6	68.7	53.9	37.0–51.5	-16.4; -22.2	-336.1; -337.8
**N4P**	-2.1; -0.3	13.6	23.4	74.9	69.1	55.2	36.5–52.0	-17.0; -21.9	-325.4; -336.0; -337.8
**N5P**	-1.4; -0.3	13.7	23.7	74.1	69.0	55.8	36.0–52.0	-16.8; -22.0	-336.0; -337.5
**N6P**	-1.5; -0.2	13.9	23.2	74.7	69.3	55.7	38.0–53.0	-16.9; -22.2	-325.4; -336.0; -337.5

### IR spectroscopy

**Fig 2:**
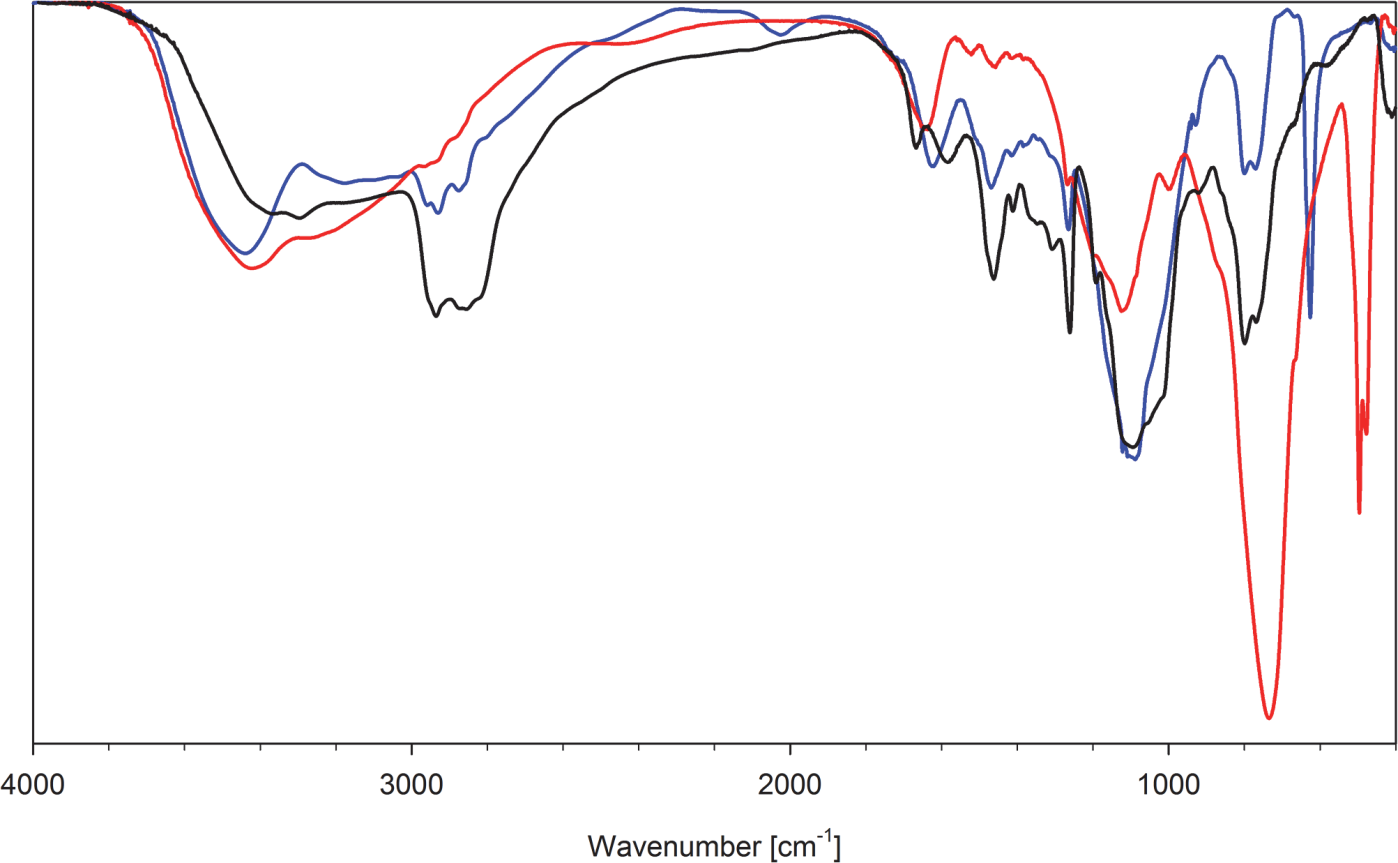
FT-IR spectra of parent polymer (N6; solid black line), N4 perchlorate (N6HCl; solid blue line) and N4 hexafluorophosphate(V) (N6HP; solid red line).

All FT-IR spectra of the synthesized resins show the signals characteristic of polysiloxane matrix, i.e. 1255–1265, 1405–1415 (δ (Si-)CH_3_), 800–810, 760–770 (γ(Si-)CH) and 1000–1100 cm^-1^ (ν Si-O-Si). These values are very close to those of the parent, neutral polymers [27]. Significant changes are observed for the absorption bands related to podand moieties. The spectra of protonated polymers show a signal at ca. 1630 cm^-1^ (δ NH_2_^+^, δ NH_3_^+^) and the bands corresponding to δ CH vibration (1430–1470 cm^-1^). The protonation also changes the shape of the signals in the ν N-H and ν O-H regions. The ν N-H absorption is shifted to smaller wavenumbers (2500–3200 cm^-1^), while the ν O-H signal is sharpened and appears at ca. 3480 cm^-1^. Counter ions give the signal at 1115 and 625 cm^-1^ (perchlorate) or 740 and 505 cm^-1^ (hexafluorophosphate). The exemplary spectra are presented in Fig 2.

**Fig 3:**
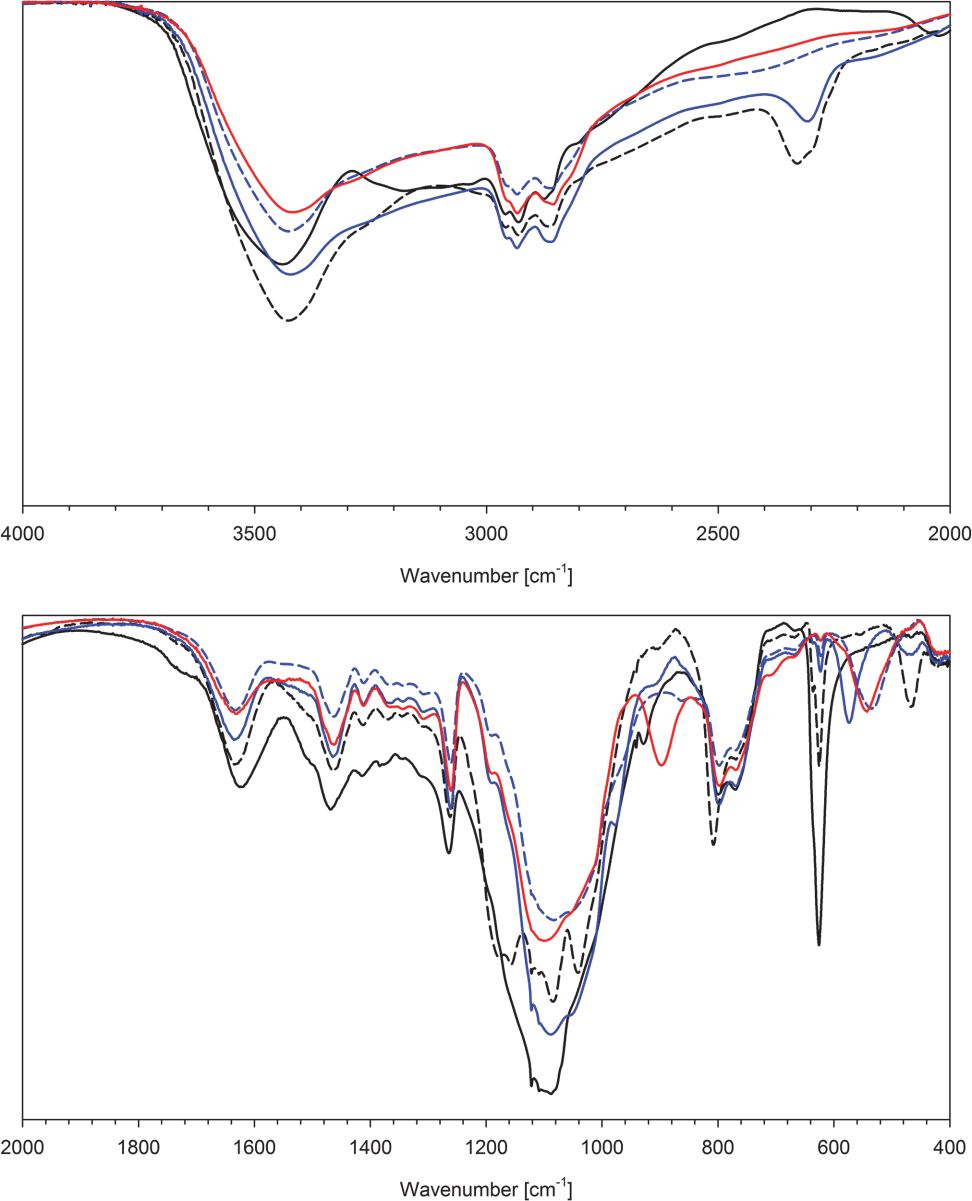
FT-IR spectra of N6HCl (solid black line) and this resin after ion-exchange with: H_2_PO_2_^-^ (dashed black line), HPO_3_^2-^ (solid blue line), PO_4_^3-^ (dashed blue line) and P_2_O_7_^4-^ (solid red line).

**Fig 4:**
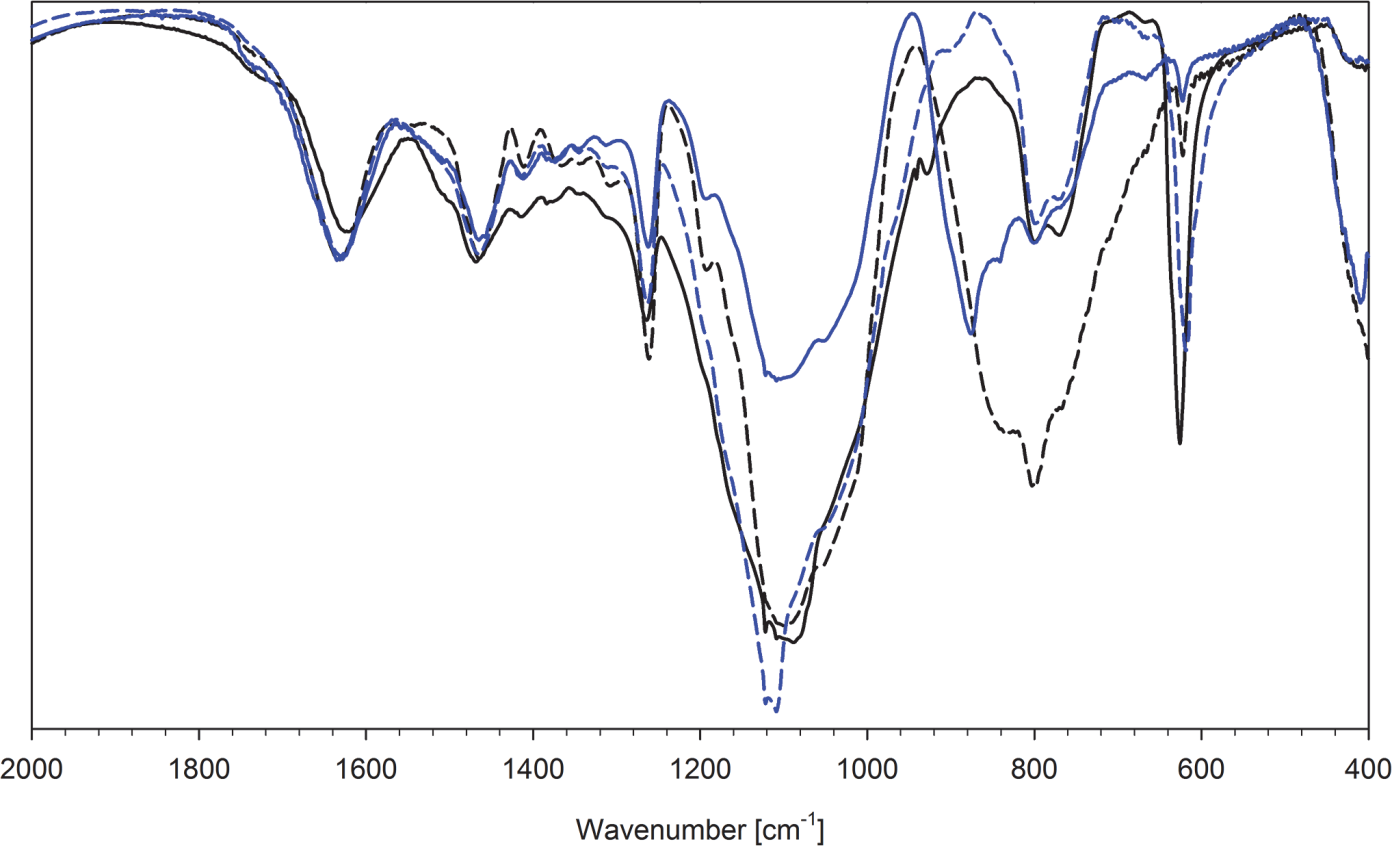
FT-IR spectra of N6HCl (solid black line) and this resin after ion-exchange with: HAsO_4_^2-^ (dashed black line), SeO_4_^2-^ (solid blue line) and SO_4_^2-^ (dashed blue line).

**Fig 5:**
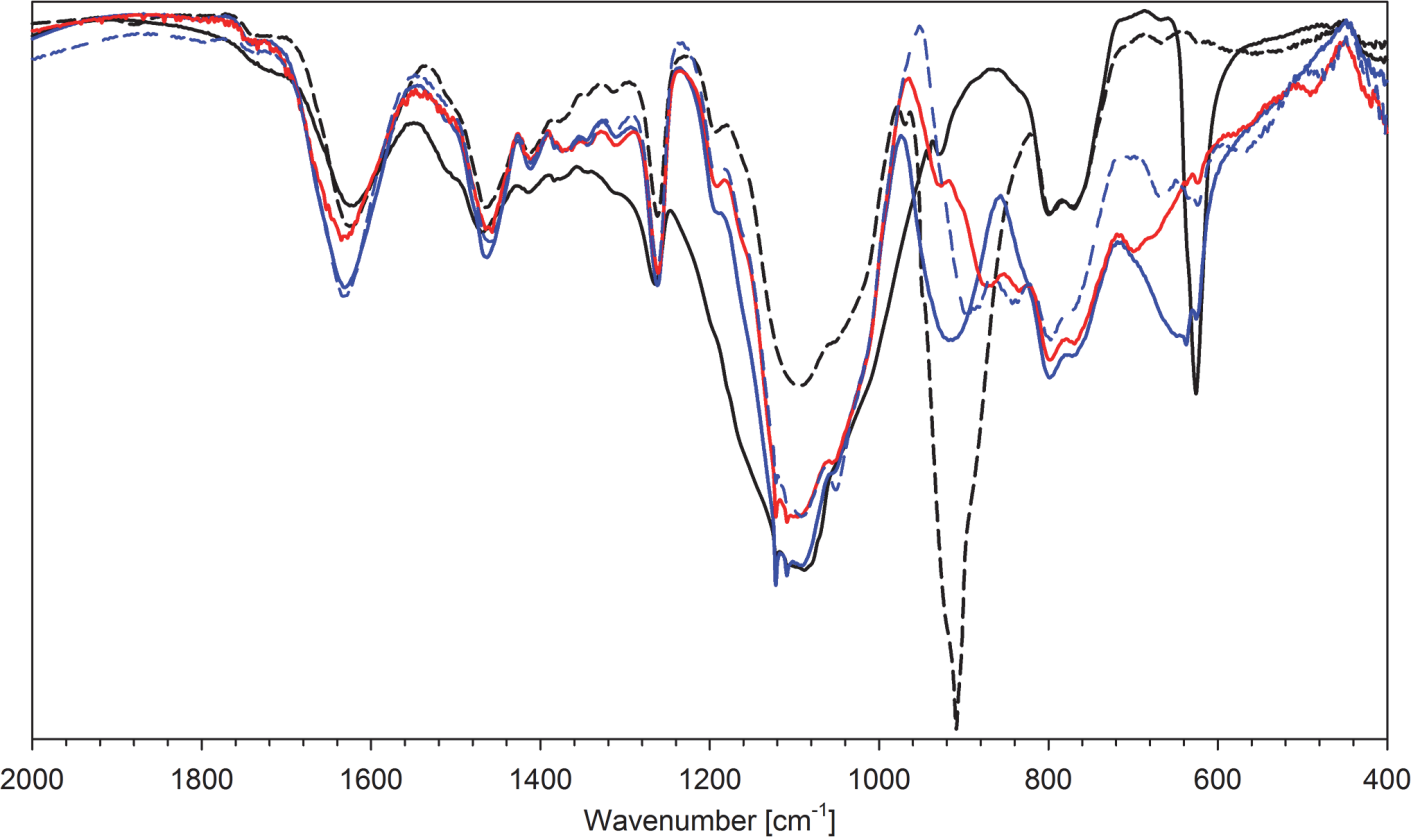
FT-IR spectra of N6HCl (solid black line) and this resin after ion-exchange with: ReO_4_^-^ (dashed black line), VO_3_^-^ (solid blue line), MoO_4_^2-^ (dashed blue line), WO_4_^2-^ (solid red line).

Anion exchange does not affect significantly the bands of the organic part of the studied polymers. The strongest shifts are observed for ν N-H and ν O-H, due to different hydrogen bond interactions between NH and OH protons and bonded anions. Bonding of the anion results in weakening or even disappearance of the signals assigned to Cl-O (or P-F) bonds and appearance of new absorption bands related to the complexed anions. The wavenumbers of the signals observed for bonded anions are collected in Table 5. The exemplary IR spectra are presented in Fig 3–5.

**Table 5:** Characteristic IR absorption bands of ions adsorbed on the resins studied.

Anion	Bands observed [cm^-1^]
PO_4_^3-^	1045 (ν P-O), 540 (δ P-O)
HPO_3_^2-^	2316 (ν P-H), 1058 (δ P-H, P-H), 1025, 1004 (ν P-O), 578 (δ _s_ P-O), 507 (δ _a_ P-O)
H_2_PO_2_^-^	2336 (ν_s_ P-H), 2304 (ν_as_ P-H), 1185 (ν_as_ P-O), 1161 (δ P-H), 1091 (ω P-H), 1047 (ν_s_ P-O), 810 (ρ P-H), 473 (δ P-O)
P_2_O_7_^4-^	1115 (ν P-O), 903 (ν_as_ P-O-P), 551 (δ P-O)
P_3_O_9_^3-^	1130 (ν P-O), 900 (ν_as_ P-O-P), 540 (δ P-O)
SO_3_^2-^	983 (ν S-O),
SO_4_^2-^	1122 (ν_as_ S-O), 615 (ν_s_ S-O), 420 (δ S-O)
SeO_3_^2-^	751 (ν Se-O)
SeO_4_^2-^	881 (ν Se-O), 414 (δ Se-O)
TeO_3_^2-^	735 (ν Te-O), 710 (ν Te-O), 630 (ν Te-O)
TeO_4_^2-^	742 (ν Te-O), 670 (ν Te-O), 410 (δ Te-O)
AsO_2_^-^	919 (ν As-O), 416 (δ As-O)
HAsO_4_^2-^	844 (ν As-O), 806 (ν As-O), 742 (ν As-O), 404 (δ As-O)
ReO_4_^-^	909 (ν_s_ As-O), 891 (ν_as_ As-O)
MoO_4_^2-^	933 (ν Mo-O; Mo_7_), 896 (ν Mo-O; Mo_7_), 830 (ν Mo-O; Mo_1_)
WO_4_^2-^	882 (ν_s_ W-O), 841 (ν_as_ W-O)
CrO_4_^2-^	922 (ν_s_ Cr-O), 890 (ν_as_ Cr-O), 765 (ν Cr-O-Cr)
VO_3_^-^	924 (ν_s_ VO_2_), 832 (ν_as_ VO_2_), 655 (ν_as_ VOV)
VO_4_^3-^	835 (ν V-O)
ClO_4_^-^	1115 (ν Cl-O), 625 (δ Cl-O)
ClO_3_^-^	973 (ν_s_ Cl-O), 918 (ν_as_ Cl-O), 617 (δ Cl-O)
BrO_3_^-^	795 (ν Br-O)
NO_3_^-^	1372 (ν_as_ N-O), 835 (δ N-O)
NO_2_^-^	1262 (ν_as_ N-O), 827 (δ N-O)
PF_6_^-^	740 (ν_a_ P-F), 505 (ν_as_ P-F)

Several important conclusions could be drawn on the basis of the IR measurements for bonded anions. The first is that the pyrophosphate (P_2_O_7_^4-^) and trimetaphosphate (P_3_O_9_^3-^) ions do not undergo hydrolysis upon complexation, since the characteristic vibrations, related to P-O-P (ca. 900 cm^-1^) are observed. On the other hand, the sorption of molybdate(VI) ions (MoO_4_^2-^) causes its partial polymerisation and formation of heptamolybdate(VI) species (Mo_7_O_24_^6-^). The IR spectra of the Mo(VI) oxoanions adsorbed on the studied resins show the bands characteristic of MoO_4_^2-^ (830 cm^-1^) as well as Mo_7_O_24_^6-^ (896 & 930 cm^-1^). The signals of both forms have similar intensities. The band at 765 cm^-1^ observed for chromate-loaded resins indicates that the adsorbed ion forms a dimer, i.e. dichromate anion.

The X-O stretching bands of all studied oxoanions bonded to resins are quite broad and composed of many close lying signals. They are distinctly broader than those reported for anhydrous inorganic or tetraalkylammonium salts. The observed effect is a result of the possibility of realisation of different modes of hydrogen bonding between oxoanion adsorbed and the O-H and N-H donors (hydroxyl and ammonium moieties of the resin as well as water molecules co-adsorbed).

### Thermal analysis

**Fig 6:**
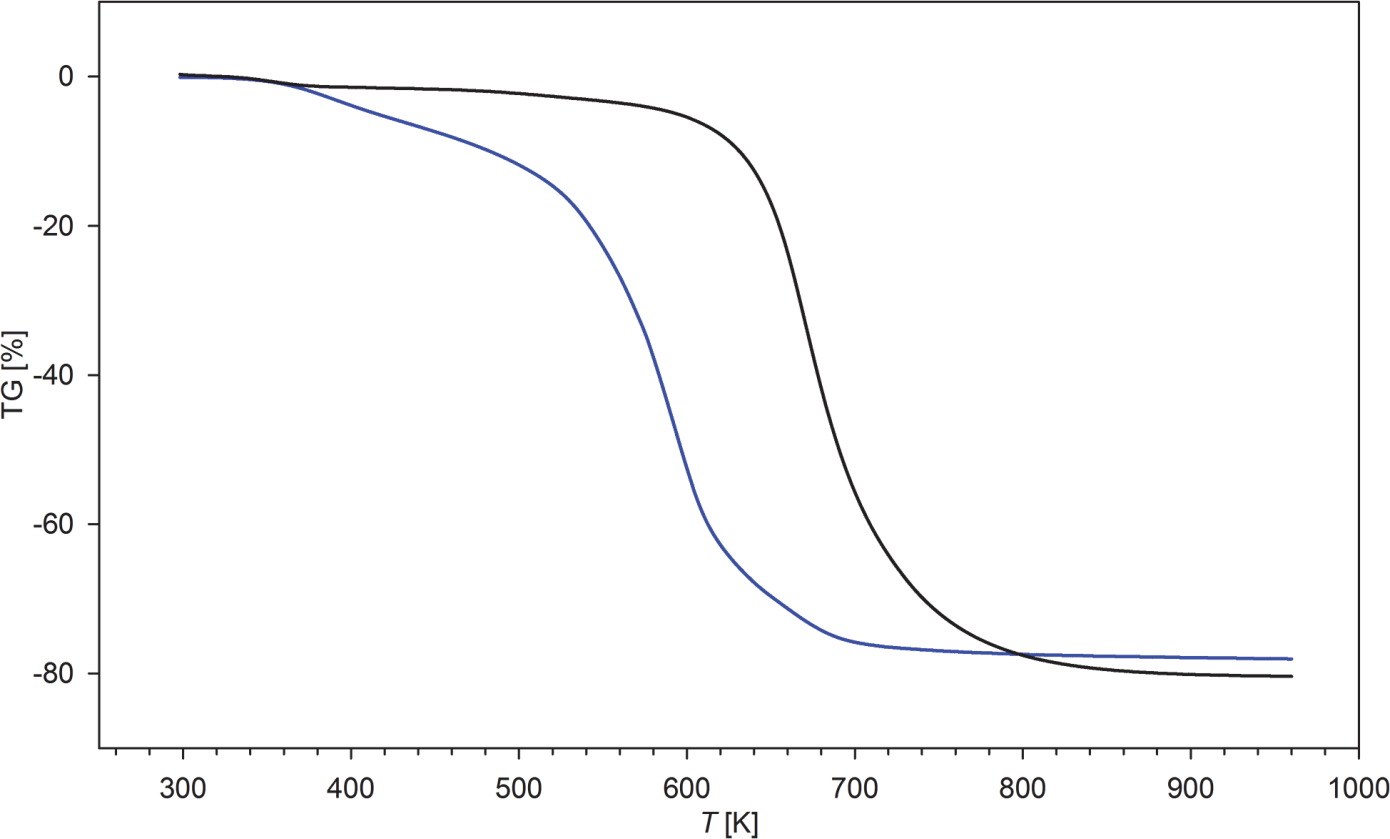
TG curves of N4 (solid black line) parent polymer and its hexafluorophosphate(V) salt (N4HP; solid blue line).

**Fig 7:**
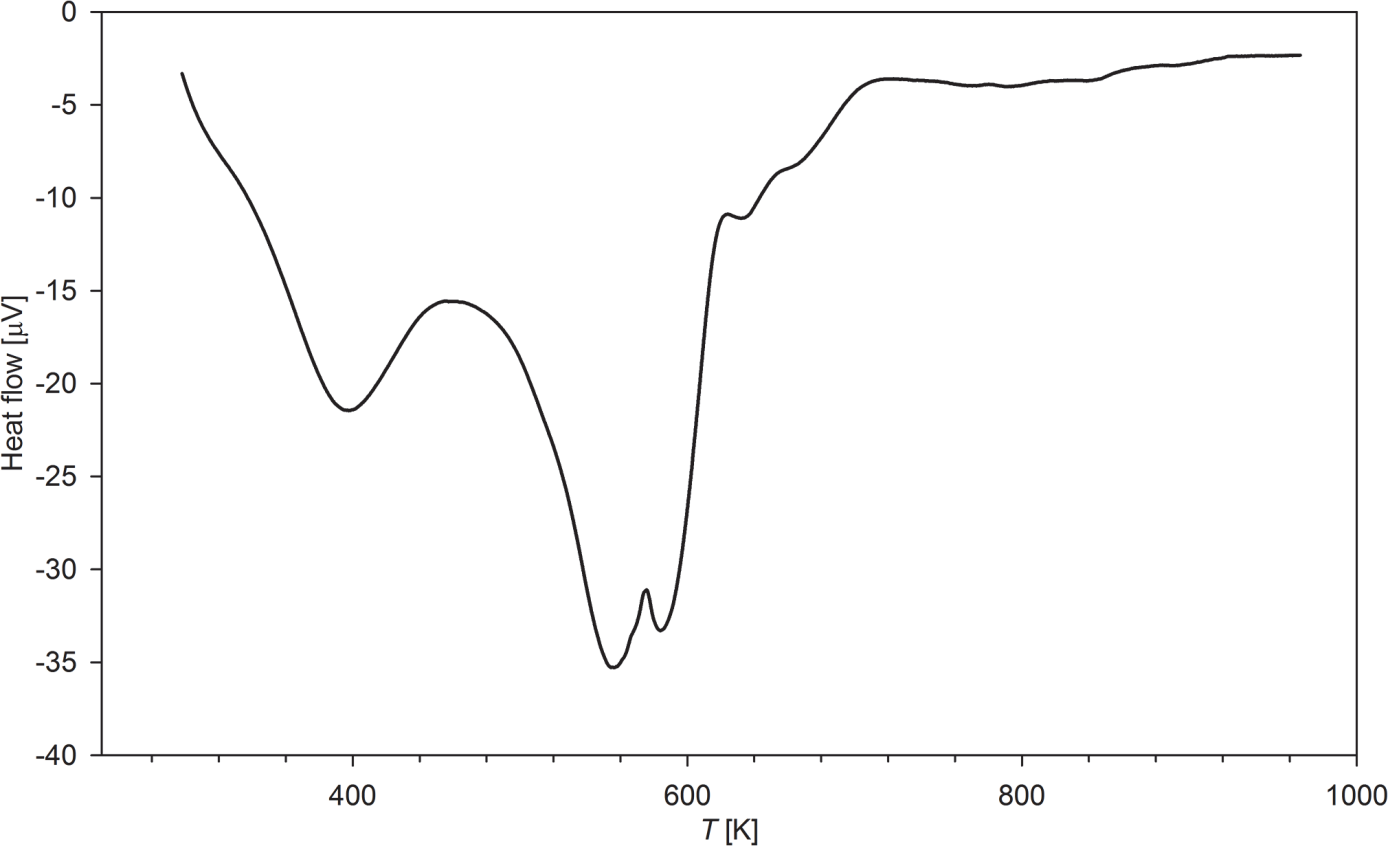
DSC curve of N4HP resin.

The thermograms of the selected polymers studied are presented in Fig 6. The perchlorate forms of the synthesized ionic resins were not studied by TG and DSC because of the violent, explosive decomposition above 250°C. Contrary, PF_6_^-^ salts decompose less rapidly, however at lower temperatures than that of the parent, neutral polymers which decompose in the temperature range 325–525°C [27]. Decomposition of ammonium polymers studied consists of several steps. Firstly, between 120 and 150°C, an endothermic process accompanied by an insignificant mass loss (ca. 5–10%) is observed. It is assigned to desorption of water adsorbed in the resin. The major decomposition is observed in the temperature range 200–350°C with two peaks on DSC curve, the first at 270°C (mass loss ca. 25–40%) and the second at 310°C (mass loss of 60–65%). Over 350°C the final decomposition step is observed, resulting in the next substantial loss of mass (up to 75%). It ends at ca. 400°C. As the low-temperature processes should be assigned to pyrolysis of organic part of the polymer, the high temperature ones corresponds to evolution of PF_5_, HF and other products of PF_6_^-^ decomposition. As for **N2**-**N6** polymers, the mass loss of ionic resins increases with the number of ethyleneamine units. The residual mass varies from 45% (**N2HP**) to 32% (**N6HP**). The residual material contains mainly Si, P, O and traces of F and C. All processes observed on heating of the samples are endothermic (Fig 7). None of the observed processes is reversible, therefore the studied resins do not undergo a glass-transition in the studied temperature ranges. The observed differences in thermal stability between the parent neutral polymers and its protonated derivatives are a result of thermal instability of ammonium salts which undergo elimination reaction, with production of R_3_NH^+^ and C = C double bond.

### Anion exchange

All polymers studied are able to exchange anions. The binding capacity of the resins depends on its structure and the anion character. Although the Misak model (Eq 7) is much more adequate for describing the ion exchange [33,34], for the anions originating from weak, polyprotic acids, the determination of the major ionic species adsorbed on a resin is difficult.

$$\frac{C_{A}}{q_{A}}=\frac{\left(\frac{x}{y}\right)C_{A}}{Q}+\frac{C_{B}^{\frac{x}{y}}}{QK^{\frac{1}{y}}}$$ 7

where *C*_*A*_, *C*_*B*_ are the equilibrium concentrations of the adsorbate (A) and counter ion (B) released to the solution, *q*_*A*_—the amount of ions bonded at the concentration *C*_*A*_, *Q* maximum amount of ions A bonded, *x*, *y*—valence of the ions A and B, *K*—equilibrium constant of the exchange reaction.

Therefore, the maximum amount of the ions bonded (*Q*, mmol of anion per 1 g of polymer) and the binding equilibrium constant *K*, were calculated assuming that the adsorption process was described by the Langmuir model (with Boyd modification; Eq 8), which is widely used in ion exchange studies [35]:

$$\frac{C_{A}}{q_{A}}=\frac{C_{A}}{Q}+\frac{C_{B}}{QK}$$ 8

Calculated *K* and *Q* values and are given in Table 6.

**Table 6:** Parameters of anions adsorption by the selected polymers studied in water at 298 K (pH = 5.0).

Anion	Adsorption parameters: *q*_*m*_ (mmol g^-1^; ± 0.02) and *K* (± 0.05)											
	N2Cl		N2P		N4Cl		N4P		N6Cl		N6P	
	*q*_*m*_	*K*	*q*_*m*_	*K*	*q*_*m*_	*K*	*q*_*m*_	*K*	*q*_*m*_	*K*	*q*_*m*_	*K*
PF_6_^-^	3.99	0.14	3.50	1.00	4.99	0.13	4.20	1.00	5.11	0.15	4.54	1.00
ClO_4_^-^	4.25	1.00	3.70	7.21	5.20	1.00	4.36	7.24	5.70	1.00	5.19	6.80
F^-^	2.15	<0.02	1.93	0.11	2.31	<0.02	1.98	0.10	2.10	<0.04	1.75	0.15
Cl^-^	4.22	0.11	3.65	0.77	5.13	0.13	4.33	0.90	5.42	0.12	4.83	0.86
Br^-^	4.20	0.11	3.61	0.75	5.00	0.19	4.21	1.26	5.20	0.15	4.67	1.07
I^-^	3.80	0.10	3.43	0.75	5.01	0.17	4.27	1.11	5.45	0.15	4.95	0.94
ClO_3_^-^	4.00	0.67	3.55	4.88	5.04	0.73	4.20	5.10	5.49	0.67	4.95	4.40
BrO_3_^-^	3.95	0.58	3.39	4.11	4.98	0.64	4.20	4.52	5.26	0.63	4.69	4.27
NO_2_^-^	2.03	0.11	1.72	0.80	2.80	0.10	2.33	0.27	3.88	0.07	3.42	0.30
NO_3_^-^	4.07	0.72	3.57	5.10	4.89	0.80	4.15	5.80	5.15	0.85	4.50	5.72
H_2_PO_2_^-^	3.34	0.53	2.96	3.75	4.02	0.59	3.38	4.14	4.44	0.65	4.05	4.33
HPO_3_^2-^	3.20	0.90	2.77	6.41	3.97	1.00	3.35	7.00	3.52	0.99	3.23	6.69
PO_4_^3-^	3.40	2.06	2.94	14.77	4.04	2.18	3.44	15.99	3.95	2.16	3.53	14.05
P_2_O_7_^4-^	1.89	3.15	1.72	23.50	2.50	3.33	2.07	25.02	2.58	3.80	2.29	25.20
P_3_O_9_^3-^	1.86	2.98	1.68	21.00	2.27	3.31	1.93	25.13	2.40	3.63	2.13	24.17
AsO_2_^-^	1.02	<0.02	0.81	<0.05	1.75	<0.02	1.50	0.11	2.02	<0.02	1.81	<0.10
HAsO_4_^2-^	2.78	3.40	2.49	24.39	3.68	3.45	3.13	23.54	3.92	3.34	3.47	22.12
SO_3_^2-^	1.46	<0.02	1.23	<0.05	2.40	<0.04	2.00	0.40	2.84	0.09	2.52	0.66
SO_4_^2-^	2.99	3.12	2.56	22.79	3.69	3.05	3.07	21.46	3.95	3.17	3.54	21.49
SeO_3_^2-^	1.32	<0.04	1.12	0.15	2.22	0.05	1.90	0.66	2.90	0.08	2.69	0.50
SeO_4_^2-^	3.03	3.94	2.66	27.99	3.99	4.20	3.35	30.05	4.07	4.13	3.62	27.23
TeO_3_^2-^	1.72	0.11	1.50	0.68	3.08	0.09	2.67	0.87	3.80	0.14	3.37	1.00
TeO_4_^2-^	0.99	<0.02	0.77	<0.05	1.70	<0.02	1.34	0.23	2.07	<0.02	1.89	0.17
VO_3_^-^	2.47	2.31	2.22	16.41	3.51	2.63	2.99	19.03	3.91	2.56	3.60	17.07
VO_4_^3-^	2.86	5.58	2.50	41.60	3.74	5.73	3.21	44.08	3.97	5.67	5.05	37.51
CrO_4_^2-^	2.45	2.99	2.24	22.06	3.49	3.15	2.86	24.23	3.61	3.22	3.27	21.27
MoO_4_^2-^	2.22	3.09	2.00	21.45	3.13	3.30	2.60	25.38	3.48	3.19	3.09	21.24
WO_4_^2-^	2.16	3.41	1.84	21.95	2.97	3.39	2.50	26.08	3.24	3.45	2.97	22.50
MnO_4_^-^	2.97	0.35	2.55	2.06	3.96	0.43	3.36	3.31	4.00	0.39	3.62	2.31
ReO_4_^-^	3.07	5.08	2.69	33.10	4.03	5.17	3.36	39.77	4.31	5.22	3.84	35.00

**Fig 8:**
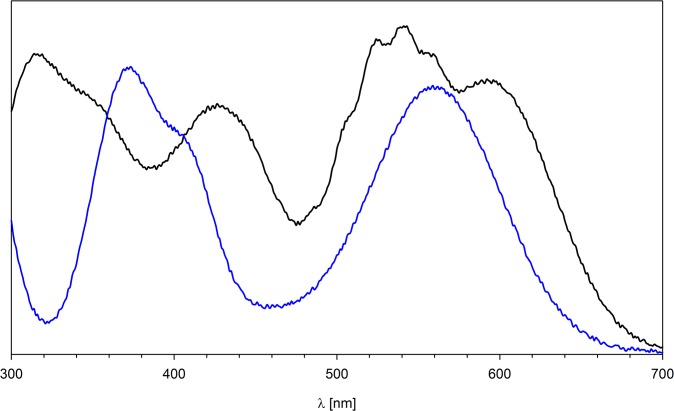
UV-Vis reflectance spectra of N4HP resin after exchange with MnO_4_^-^ (solid black line) and CrO_4_^2-^ (solid blue line).

For the studied weak base anion exchange resins, the affinity of monovalent halogen anions to polymers is very low. All of them interact with the resins studied weaker than PF_6_^-^ and ClO_4_^-^. The interaction of oxoanions depends on their valence and the strength of the parent acid. The monovalent oxoanions of strong acids (ClO_4_^-^, BrO_4_^-^, ReO_4_^-^ etc.) show similar affinity to the resin, however the larger anions (e.g. ReO_4_^-^) bind stronger to the less cross-linked polymers of a more flexible structure (**N5H**, **N6H**). The monovalent anions of very weak acids (AsO_2_^-^ or As(OH)_2_O^-^) practically do not exchange with ClO_4_^-^ and only slightly with PF_6_^-^ ions. This is a result of several cooperative effects. The first is that at pH = 5.0 they exist in a neutral form, so they do not bind to the protonated resin via electrostatic interaction. Moreover, the oxygen atoms of arsenate(III) acid are poorer hydrogen bond acceptors than those of perchlorate anion. Comparing the ions containing the same central atom, it is clearly seen that the affinity to the resin increases with increasing oxidation state of the central atom, e.g. the *K* values for SO_3_^2-^ or SeO_3_^2-^ are under one-tenth of those of SO_4_^2-^ or SeO_4_^2-^. This is related to increasing acid strength and, in consequence, the domination of multiple charged species in the solution. At pH = 5.0 the selenous or sulfurous acids exists mainly as monovalent, HXO_3_^-^ anions. They make weaker electrostatic interactions with N^+^ centres than the multiple charged ions. Moreover, the increasing number of oxygen atoms in the anions containing central atom of high oxidation state, stabilises adducts due to the formation of higher number of cooperative hydrogen bonds with NH and OH donors from the resin. The same effects are observed for the series of phosphates(V). The orthophosphate ion (PO_4_^3-^) is less effectively adsorbed than diphosphate(V) (pyrophosphate, P_2_O_7_^4-^) or trimetaphosphate(V) (P_3_O_9_^3-^) ones. At pH = 5.0 the major form of phosphoric(V) acid is the monovalent anion (H_2_PO_4_^-^), since in the same conditions diphosphoric(V) acid forms double charged ion (H_2_P_2_O_7_^2-^) and trimetaphosphoric acid dissociates almost completely (the molar fraction of P_3_O_9_^3-^ is ca. 0.999). The effect of the number of possible hydrogen bonds is clearly seen for metavanadate(V) and orthovanadate(V). At pH = 5.0, they both exist as monovalent ions (molar fraction of H_2_VO_4_^-^ and VO_3_^-^ is ca. 0.94), however for metavanadate, *K* values are distinctly lower. The strong oxidizing anions (MnO_4_^-^, CrO_4_^2-^) undergo partial reduction after adsorption. Fig 8 presents the diffusion reflectance UV-Vis spectra of **N6HCl** loaded with permanganate and (di)chromate ions. Besides the absorption of Mn(VII) or Cr(VI) (520 nm and 350 nm, respectively), the signals characteristic of MnO_2_ (430, 600 nm) and Cr^3+^ (410, 560 nm) are observed.

**Fig 9:**
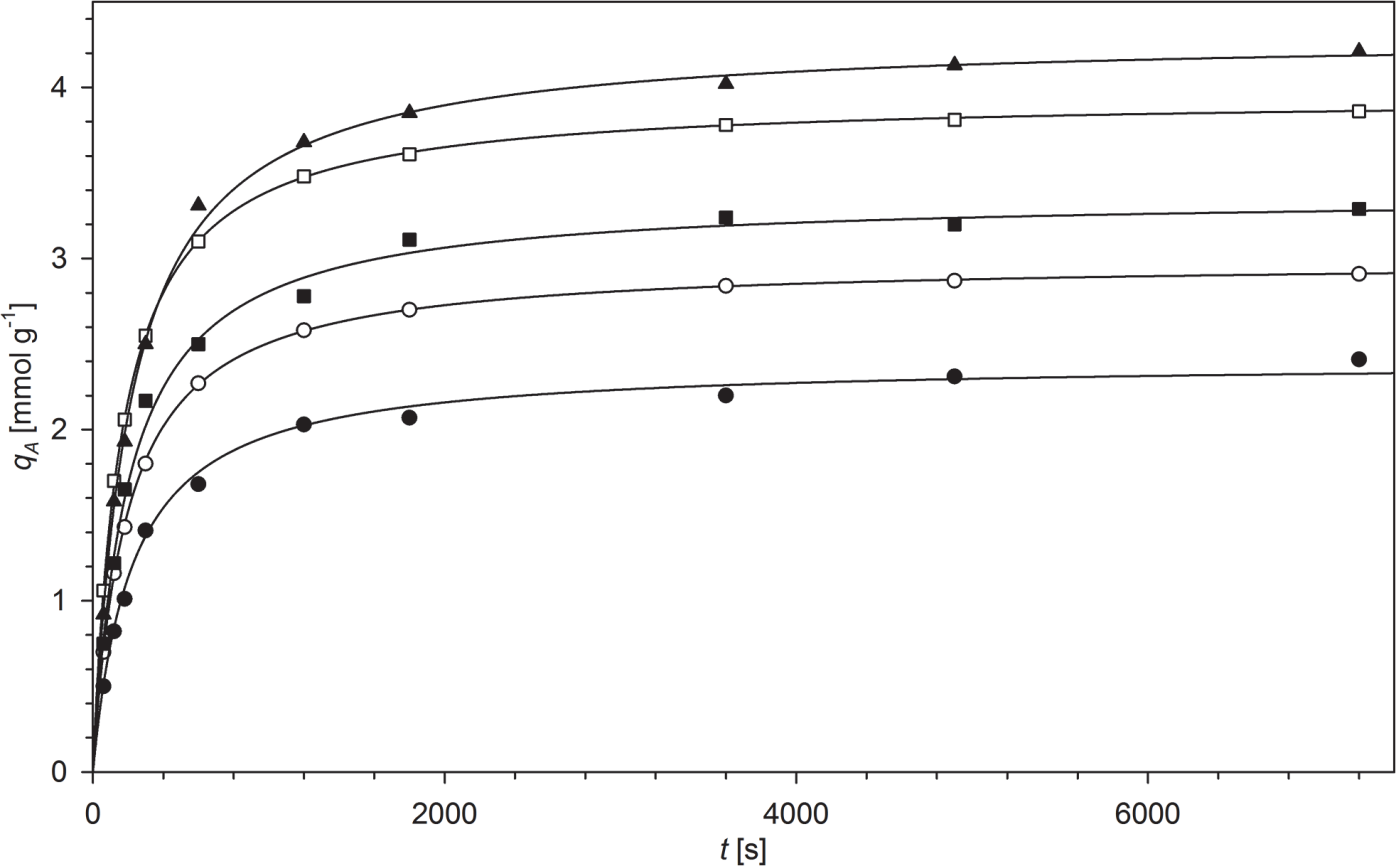
Pseudo-second order kinetic curves of anion exchange: ○ —N2HCl + SO_4_^2-^, □ —N6HCl + SO_4_^2-^, ▲ —N6HCl + ReO_4_^-^, ■ —N6HCl + TeO_3_^2-^, ● —N6HCl + P_3_O_9_^3-^.

The kinetic parameters of ion exchange were studied for selected ions. The ions undergoing reaction with the resin (MnO_4_^-^, CrO_4_^2-^) or polymerizing during adsorption (MoO_4_^2-^) were excluded from the study. Also some ions which were very weakly bonded by the studied materials (e.g. F^-^, AsO_2_^-^, SO_3_^2-^) were not investigated. The kinetics of the reaction was measured for four resins, two perchlorate ones (**N2HCl** and **N6HCl**) and their PF_6_^-^ analogues (**N2HP**, **N6HP**). The exemplary kinetic curves are presented in Fig 9. The kinetic parameters are collected in Table 7. The equilibrium of the reaction is reached quite quickly, in less than 60 min., depending on the anion adsorbed. The adsorption process was analysed using two kinetic models, i.e. pseudo-first order and pseudo-second order [36]. The experimental data were much better reproduced by the second one. Although this simplified approach does not take the sorption mechanism into account, it is widely used for description of the sorption kinetics. Therefore, the presented results should be used only for the comparison of the sorption kinetics properties of the studied materials with literature data and have no physical meaning. The exchange was distinctly slower for **N2**-derived resins than for **N6**-derived ones. This is a consequence of the slower diffusion of ions in the polymer matrix cross-linked with shorter polyamine chains. The differences observed are more distinct for large ions (e.g. ReO_4_^-^) than for those of small radii (Cl^-^), whose diffusion is less hindered by the close distances between the polysiloxane chains. There is no significant difference in the process kinetics between the perchlorate and hexafluorophosphate forms of the exchangers.

**Table 7:** The kinetics of ion exchange by the polymers studied in water (278 K; pH = 5.s0)—pseudo-second order rate constants.

Anion	Kinetic parameters—rate constant (×1000 g (mmol s)^-1^); ± 0.1			
	N2HCl	N2HP	N4HCl	N4P
Br^-^	6.8	6.8	7.4	7.2
I^-^	6.0	5.9	6.9	7.0
ClO_3_^-^	5.2	5.3	6.0	6.0
BrO_3_^-^	4.8	4.5	5.6	5.8
NO_3_^-^	6.7	6.6	7.2	7.0
H_2_PO_2_^-^	5.0	4.9	6.0	6.0
HPO_3_^-^	5.0	5.0	5.9	6.1
PO_4_^3-^	5.1	4.9	6.2	6.0
P_2_O_7_^4-^	3.5	3.4	4.7	4.6
P_3_O_9_^3-^	3.0	3.0	4.5	4.5
HAsO_4_^2-^	4.0	4.2	5.3	5.2
SO_4_^2-^	5.2	5.1	6.1	6.1
SeO_4_^2-^	4.1	4.3	5.4	5.4
TeO_3_^2-^	3.3	3.2	5.0	5.2
VO_3_^-^	3.3	3.0	4.6	4.8
VO_4_^3-^	3.0	3.1	4.4	4.7
WO_4_^2-^	3.4	3.1	4.4	4.3
ReO_4_^-^	2.8	2.6	4.2	4.1

**Fig 10:**
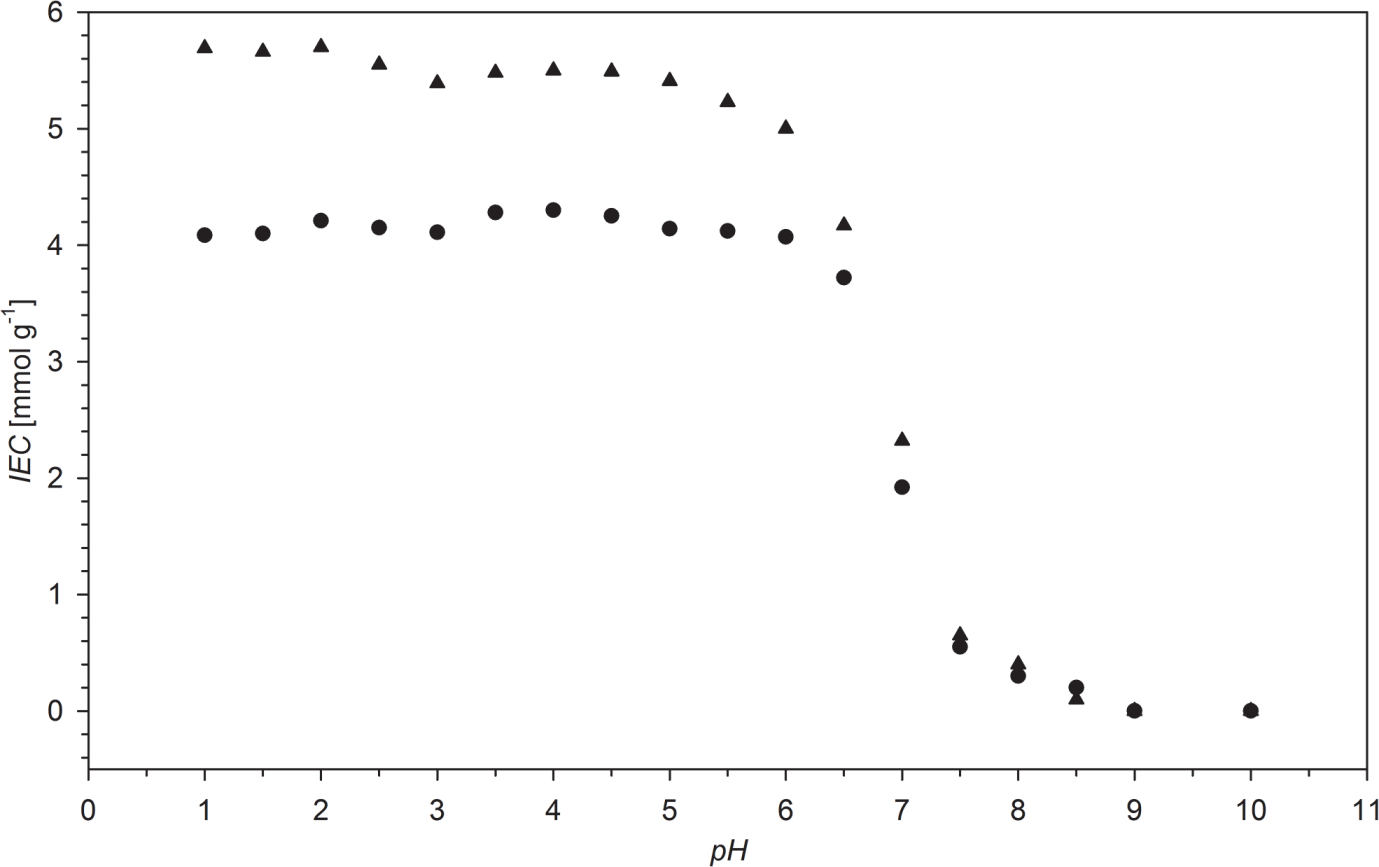
Ion-exchange capacity (IEC) of N2HCl (●) and N6HCl (■).

**Fig 11:**
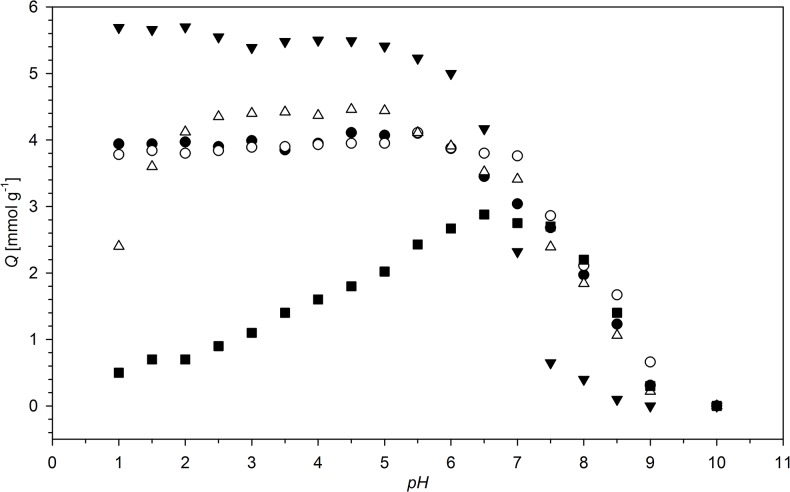
The pH dependence of maximal amount of ion bonded (*Q*) for N6HCl and: ▼ —ClO_4_^-^, ○ —SO_4_^2-^, ● —SeO_4_^2-^, Δ —H_2_PO_2_^-^, ■ —AsO_2_^-^.

The pH dependence of the ion-exchange capacity (IEC) of the studied resins is shown in Fig 10. As expected, IEC is almost constant below pH = 5.5. Above this value, IEC drops and reaches 0 at pH > 8. This is a result of deprotonation of NH^+^ centres of the resin. Above pH = 8, the resins exist in neutral, amine form. Any binding of anions in the pH range 8–14 is a physisorption or a result of NHO and OHO hydrogen bonding between resin heteroatoms and anion oxygen atoms. The influence of pH of the solution on the maximum amount of ions bonded (*Q*) is presented in Fig 11. At low pH values (pH < 3) the anions of strong acids (sulphate, selenate, perchlorate etc.) are preferably adsorbed. Anions of strong acids exist in ionic form even at low pH. The uptake of these anions rise insignificantly with increasing pH, these changes are more distinct for multivalent anions. As the second and subsequent dissociation constants of polyprotonic acids are smaller than the first one, the increasing pH value results in the rise of the population of double (and triple) ionized species, which interact stronger with the protonated resin. Contrary, the anions of weak acids show high increase in the affinity to the resin with decreasing solution acidity. At low pH, these acids exist as non-ionized molecules or the molar ratios of ionic forms are very low. The increase in pH results in ionisation of weak acids and formation of anionic species, which show affinity to the resin. The anion of the weakest acid studied (AsO_2_^-^) adsorbs at pH > 8 and the formed adduct is stabilised by hydrogen bonds.

### Sorption/regeneration experiments

**Fig 12:**
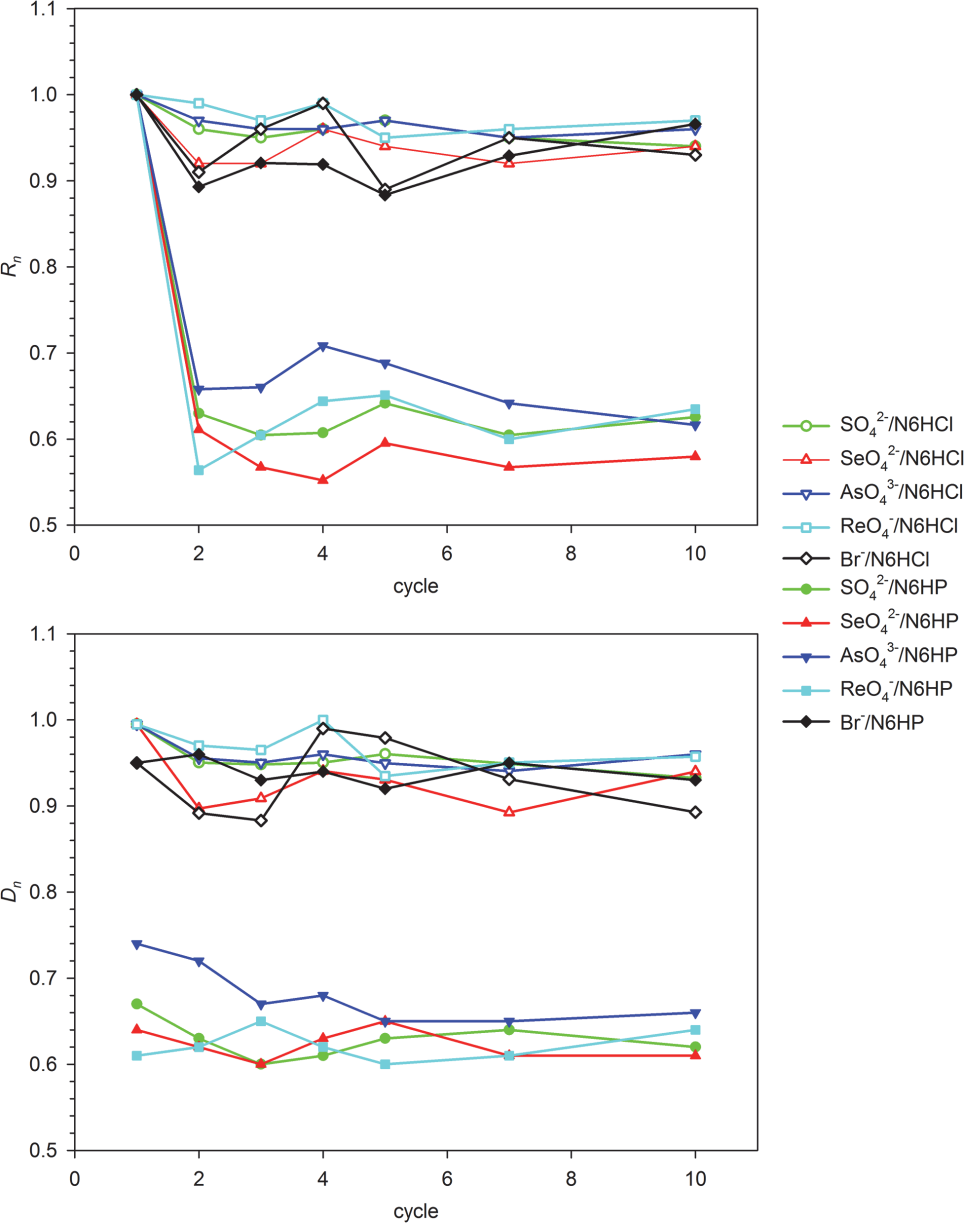
Anion retention (*R_n_*) and release (*D_n_*) dependence on the number of sorption-regeneration cycles (base/acid regeneration procedure).

**Fig 13:**
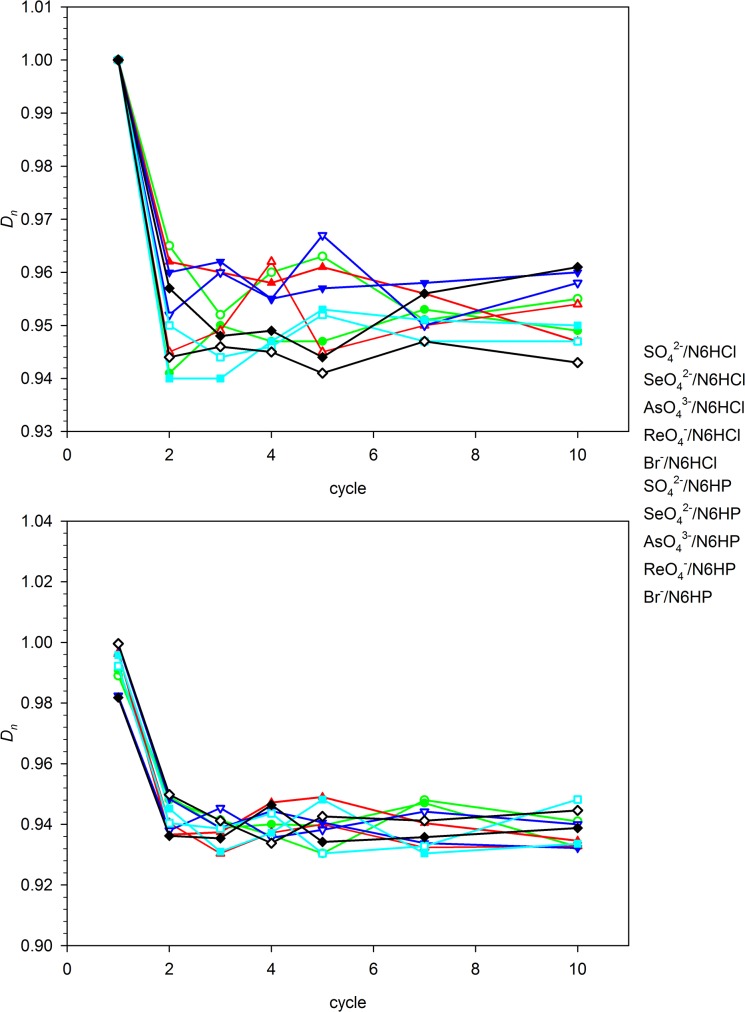
Anion retention (*R_n_*) and release (*D_n_*) dependence on the number of sorption-regeneration cycles (brine regeneration procedure).

Sorption-regeneration experiments were carried out for **N6HCl**, **N6HP** and selected ions: Br^-^, SO_4_^2-^, SeO_4_^2-^, ReO_4_^-^ and HAsO_4_^2-^ (these formulas correspond to the composition of salts used as an anion sources, not to the form of the anions existing in solution or bonded to the polymer after sorption, which may be different due to protolytic equilibria). The results of these experiments are shown in Fig 12 and 13. Fig 12 presents the efficiency of two-step regeneration, i.e. desorption of bonded ion in NaOH solution followed by re-protonation of the polymer material. Synthesized material shows excellent reversibility of the exchange properties and high stability. Small (ca. 5%) decrease in the sorption efficiency (*R*) is shown in the first sorption-regeneration cycle, but the further amounts of exchanged ions are the same up to the tenth cycle. Over 97% of the ions adsorbed are released during treatment with NaOH. The regeneration with concentrated salt (NaClO_4_ or NaPF_6_) solutions gives somewhat different results (Fig 13). Since the regeneration with NaClO_4_ almost restores the starting sorption ability of the materials studied, the regeneration with NaPF_6_ is much less effective. The recovery of oxoanions of strong acids, showing high affinity to the resins studied, in this regeneration method is ca. 60–70%. This is a result of much stronger bonding of these ions than that of a PF_6_^-^ anion, which does not form hydrogen bonds with the polymers studied. For simple anions (e.g. Br^-^), having lower affinity to the resin than PF_6_^-^, the *D*_*n*_ parameter is over 0.9.

### Conclusions

It has been shown that the studied synthetic weak-base anion exchangers show excellent ion-exchange properties. They have high ion exchange capacity (2–7 mmol g^-1^), much higher than those of the most commercially used anion-exchange resins (0.5–2 mmol g^-1^) and comparable to those reported for modern, recently reported adsorbents [28,37]. The materials studied are thermally stable and could be regenerated many times without significant loss of activity. They show high affinity to strong acids-related anions, so they could be used for separation of ions containing the same atom at different oxidation states (e.g. arsenate(III) from arsenate(V)). Their excellent adsorption properties to metal oxoions make them interesting materials for separation and pre-concentration of valuable metals (e.g. vanadium or rhenium) in hydrometallurgical processes or for removal of toxic metalloids (selenium, arsenic) from drinking water.

## References

[1] Walcarius A, Mercier L (2010) Mesoporous organosilica adsorbents: nanoengineered materials for removal of organic and inorganic pollutants. Journal of Materials Chemistry 20: 4478–4511.

[2] Roundhill DM, Koch HF (2002) Methods and techniques for the selective extraction and recovery of oxoanions. Chemical Society Reviews 31: 60–67. 12108983 10.1039/b003141k

[3] Hickner MA, Herring AM, Coughlin EB (2013) Anion exchange membranes: Current status and moving forward. Journal of Polymer Science Part B: Polymer Physics 51: 1727–1735.

[4] Loganathan P, Vigneswaran S, Kandasamy J (2013) Enhanced removal of nitrate from water using surface modification of adsorbents—A review. Journal of Environmental Management 131: 363–374. 10.1016/j.jenvman.2013.09.034 24211565

[5] Boddu VM, Abburi K, Talbott JL, Smith ED, Haasch R (2008) Removal of arsenic (III) and arsenic (V) from aqueous medium using chitosan-coated biosorbent. Water Research 42: 633–642. 17822735 10.1016/j.watres.2007.08.014

[6] Pillewan P, Mukherjee S, Meher AK, Rayalu S, Bansiwal A (2014) Removal of arsenic (III) and arsenic (V) using copper exchange zeolite-a. Environmental Progress & Sustainable Energy 33: 1274–1282.

[7] Gérente C, Andrès Y, McKay G, Le Cloirec P (2010) Removal of arsenic(V) onto chitosan: From sorption mechanism explanation to dynamic water treatment process. Chemical Engineering Journal 158: 593–598.

[8] Zhu L, Zhang C, Liu Y, Wang D, Chen J (2010) Direct synthesis of ordered N-methylimidazolium functionalized mesoporous silica as highly efficient anion exchanger of Cr(vi). Journal of Materials Chemistry 20: 1553–1559.

[9] Awual MR, Jyo A, El-Safty SA, Tamada M, Seko N (2011) A weak-base fibrous anion exchanger effective for rapid phosphate removal from water. Journal of Hazardous Materials 188: 164–171. 10.1016/j.jhazmat.2011.01.092 21320748

[10] Mustafa S, Ahmad T, Naeem A, Shah K, Waseem M (2010) Kinetics of Chromium Ion Removal from Tannery Wastes Using Amberlite IRA-400 Cl− and its Hybrids. Water, Air, & Soil Pollution 210: 43–50.

[11] Leyden DE, Luttrell GH, Nonidez WK, Werho DB (1976) Preconcentration of certain anions using reagents immobilized via silylation. Analytical Chemistry 48: 67–70.

[12] Depecker G, Branger C, Margaillan A, Pigot T, Blanc S, Robert-Peillard F, et al (2009) Synthesis and applications of XAD-4-DAN chelate resin for the separation and determination of Se(IV). Reactive and Functional Polymers 69: 877–883.

[13] Kim E-M, Jeong H-J, Heo Y-J, Moon H-B, Bom H-S, Kim G-C (2004) Intratumoral Injection of ^188^Re labeled Cationic Polyethylenimine Conjugates: A Preliminary Report. J Korean Med Sci 19: 647–651. 15483337 10.3346/jkms.2004.19.5.647 PMC2816324

[14] Srikanth MV, Sunil SA, Rao NS, Uhumwangho MU, Ramana Murthy KV (2010) Ion-Exchange Resins as Controlled Drug Delivery Carriers.

[15] Pande SV, Kshirsagar MD, Chandewar AV (2011) Ion exchange resins Pharmaceutical applications and recent advancement.

[16] Barbaro P, Liguori F (2008) Ion Exchange Resins: Catalyst Recovery and Recycle. Chemical Reviews 109: 515–529. 10.1021/cr800404j 19105606

[17] Chicz RM, Shi Z, Regnier FE (1986) Preparation and evaluation of inorganic anion-exchange sorbents not based on silica. Journal of Chromatography 359: 121–130. 3015995 10.1016/0021-9673(86)80067-x

[18] Dąbrowski A, Tertykh VA, editors (1996) Adsorption on new and modified inorganic sorbents Amsterdam: Elsevier.

[19] Nishimura T, Hashimoto H, Nakayama M (2007) Removal of Selenium(VI) from Aqueous Solution with Polyamine-type Weakly Basic Ion Exchange Resin. Separation Science and Technology 42: 3155–3167.

[20] Guimarães D, Leão VA (2014) Batch and fixed-bed assessment of sulphate removal by the weak baseion exchange resin Amberlyst A21. Journal of Hazardous Materials 280: 209–215. 10.1016/j.jhazmat.2014.07.071 25151243

[21] Chassary P, Vincent T, Guibal E (2004) Metal anion sorption on chitosan and derivative materials: a strategy for polymer modification and optimum use. Reactive and Functional Polymers 60: 137–149.

[22] Guibal E, Dambies L, Milot C, Roussy J (1999) Influence of polymer structural parameters and experimental conditions on metal anion sorption by chitosan. Polymer International 48: 671–680.

[23] Guibal E, Milot C, Tobin JM (1998) Metal-Anion Sorption by Chitosan Beads: Equilibrium and Kinetic Studies. Industrial & Engineering Chemistry Research 37: 1454–1463.

[24] Hibino T (2014) Evaluation of anion adsorption properties of nanocomposite polymer hydrogels containing layered double hydroxides. Applied Clay Science 87: 150–156.

[25] Deng S, Yu G, Xie S, Yu Q, Huang J, Kuwaki Y, et al (2008) Enhanced Adsorption of Arsenate on the Aminated Fibers: Sorption Behavior and Uptake Mechanism. Langmuir 24: 10961–10967. 10.1021/la8023138 18771297

[26] Kailasam V, Rosenberg E (2012) Oxyanion removal and recovery using silica polyamine composites. Hydrometallurgy 129–130: 97–104.

[27] Gierczyk B, Schroeder G, Cegłowski M (2011) New polymeric metal ion scavengers with polyamine podand moieties. Reactive and Functional Polymers 71: 463–479.

[28] Awual MR, Jyo A (2009) Rapid column-mode removal of arsenate from water by crosslinked poly(allylamine) resin. Water Research 43: 1229–1236. 10.1016/j.watres.2008.12.018 19152954

[29] Awual MR, Urata S, Jyo A, Tamada M, Katakai A (2008) Arsenate removal from water by a weak-base anion exchange fibrous adsorbent. Water Research 42: 689–696. 17959217 10.1016/j.watres.2007.08.020

[30] Awual MR, Hossain MA, Shenashen MA, Yaita T, Suzuki S, Jyo A (2013) Evaluating of arsenic(V) removal from water by weak-base anion exchange adsorbents. Environmental Science and Pollution Research 20: 421–430. 10.1007/s11356-012-0936-7 22562349

[31] Awual MR, Jyo A (2011) Assessing of phosphorus removal by polymeric anion exchangers. Desalination 281: 111–117.

[32] Fogg AG, Burgess C, Burns DT (1971) A critical study of Brilliant green as a spectrophotometric reagent: the determination of perchlorate particularly in potassium chlorate. Analyst 96: 854–857.

[33] Misak NZ (2000) Some aspects of the application of adsorption isotherms to ion exchange reactions. Reactive and Functional Polymers 43: 153–164.

[34] Misak NZ (1995) Adsorption isotherms in ion exchange reactions. Further treatments and remarks on the application of the Langmuir isotherm. Colloids and Surfaces A: Physicochemical and Engineering Aspects 97: 129–140.

[35] Liu Y (2006) Some consideration on the Langmuir isotherm equation. Colloids and Surfaces A: Physicochemical and Engineering Aspects 274: 34–36.

[36] Kumar KV, Sivanesan S (2006) Selection of optimum sorption kinetics: Comparison of linear and non-linear method. Journal of Hazardous Materials 134: 277–279. 16387428 10.1016/j.jhazmat.2005.11.003

[37] Awual MR, Hasan MM, Ihara T, Yaita T (2014) Mesoporous silica based novel conjugate adsorbent for efficient selenium(IV) detection and removal from water. Microporous and Mesoporous Materials 197: 331–338.

